# H19 lncRNA alters stromal cell growth via IGF signaling in the endometrium of women with endometriosis

**DOI:** 10.15252/emmm.201505245

**Published:** 2015-06-18

**Authors:** Sanaz Ghazal, Brett McKinnon, Jichun Zhou, Martin Mueller, Yi Men, Lihua Yang, Michael Mueller, Clare Flannery, Yingqun Huang, Hugh S Taylor

**Affiliations:** 1 Department of Obstetrics, Gynecology & Reproductive Sciences, Yale School of MedicineNew Haven, CT, USA; 2 Department of Obstetrics and Gynecology, University Hospital BernBern, Switzerland; 3 Department of Surgical Oncology, Affiliated Sir Run Run Shaw Hospital, Zhejiang University School of MedicineHangzhou, Zhejiang, China; 4 Department of Oral and Maxillofacial Surgery, State Key Laboratory of Oral Diseases, West China Hospital of Stomatology, Sichuan UniversityChengdu, Sichuan, China; 5 Obstetrics and Gynecology Department, Tangshan Gongren HospitalTangshan, Hebei, China

**Keywords:** endometrium, H19, IGF, let-7, long noncoding RNA

## Abstract

Endometriosis affects approximately 15% of reproductive aged women and is associated with chronic pelvic pain and infertility. However, the molecular mechanisms by which endometriosis impacts fertility are poorly understood. The developmentally regulated, imprinted H19 long noncoding RNA (lncRNA) functions to reduce the bioavailability of microRNA let-7 by acting as a molecular sponge. Here we report that *H19* expression is significantly decreased in the eutopic endometrium of women with endometriosis as compared to normal controls. We show that decreased H19 increases let-7 activity, which in turn inhibits *Igf1r* expression at the post-transcriptional level, thereby contributing to reduced proliferation of endometrial stromal cells. We propose that perturbation of this newly identified H19/Let-7/IGF1R regulatory pathway may contribute to impaired endometrial preparation and receptivity for pregnancy in women with endometriosis. Our finding represents the first example of a lncRNA-based mechanism in endometriosis and its associated infertility, thus holding potential in the development of novel therapeutics for women with endometriosis and infertility.

## Introduction

Endometriosis is defined as the presence of endometrial glands and stroma outside of the uterus (reviewed in Macer & Taylor, 2012). Approximately 6–15% of reproductive aged women are affected by endometriosis although the true incidence is unknown because some patients remain asymptomatic. The classic symptoms include dysmenorrhea, chronic pelvic pain, dyspareunia, irregular uterine bleeding, and infertility. In some cases, the disease can be debilitating and exert a dramatic impact on health and quality of life (reviewed in Macer & Taylor, 2012). There is a clear association between endometriosis and infertility. Some studies suggest that 25–50% of women with infertility also have endometriosis and 30–50% of women with endometriosis are infertile (Meuleman *et al*, 2009). However, despite the well-established link between endometriosis and infertility, the mechanism by which endometriosis impacts fertility is poorly understood.

The insulin-like growth factors, including insulin, IGF1, and IGF2, play important roles in mediating and modulating sex hormone-induced growth and differentiation of endometrial cells (Tang *et al*, 1994; Baker *et al*, 1996; Adriaenssens *et al*, 1999; Ivanga *et al*, 2007). Insulin exerts its function through interaction with the insulin receptor (INSR), whereas actions of both IGF1 and IGF2 are mainly mediated by the IGF1 receptor (IGF1R) (LeRoith *et al*, 1995). Of particular interest is the fact that IGF1-deficient female mice are infertile and exhibit uterine hypoplasia, suggesting that IGF1 is important for uterine growth and function (Baker *et al*, 1996). *Igf1* and *Igf1r* are expressed in both the epithelium and stroma of the endometrium. Their expression levels fluctuate during the menstrual cycle with the highest levels of expression being in the late proliferative phase. Their expression is regulated, at least in part by ovarian steroids, mainly 17-beta estradiol (E2) (Tang *et al*, 1994; Baker *et al*, 1996). Activation of the PI3K/AKT and Ras/Raf/MAPK signal transduction pathways through the IGF1 action promotes uterine cell growth and proliferation (Tang *et al*, 1994; LeRoith *et al*, 1995; Ivanga *et al*, 2007). Consistently, *Igf1r* overexpression with heightened AKT activity has been detected in hyperplastic endometrium (McCampbell *et al*, 2006). However, it is not known whether the expressions of *Igf1r* and/or *Igf1* are altered in the eutopic endometrium of women with endometriosis and if so, what the underlying mechanism might be.

The H19 long noncoding RNA (lncRNA) is expressed from the imprinted gene locus that also contains the reciprocally imprinted *Igf2* gene (reviewed in Gabory *et al*, 2010). The *H19* gene encodes a 2,600-nt capped, spliced, and polyadenylated noncoding RNA that is predominantly cytoplasmic (Gabory *et al*, 2010; Monnier *et al*, 2014). In human and mouse endometrium, *H19* is expressed in a menstrual cycle-dependent fashion, with increased expression during the late proliferative phase, but the physiological significance of this expression is unclear (Ariel *et al*, 1997; Adriaenssens *et al*, 1999; Tanos *et al*, 2004; Korucuoglu *et al*, 2010). Endometrial *H19* expression is positively regulated by E2 and negatively regulated by progesterone in the mouse (Ivanga *et al*, 2007). Unlike *Igf1* and *Igf1r*, which are expressed in both the epithelial and stromal compartments (Tang *et al*, 1994), *H19* expression is confined to the endometrial stroma (Ariel *et al*, 1997; Tanos *et al*, 2004). It has been recently shown that women with unexplained infertility show decreased *H19* expression in the eutopic endometrium suggesting a direct clinical link (Korucuoglu *et al*, 2010).

We have recently found that the H19 lncRNA (called H19 herein) reduces the bioavailability of the microRNA let-7 by acting as a molecular sponge (Kallen *et al*, 2013). Let-7 inhibits target gene expression by binding to complementary sequences in the mRNA, which leads to translational repression and mRNA degradation (Roush & Slack, 2008; Fabian & Sonenberg, 2012). A limited number of genes have been identified as let-7 targets and among them is *Igf1r* (Zhu *et al*, 2011). Given that women with unexplained infertility show decreased *H19* expression in their eutopic endometrium (Korucuoglu *et al*, 2010) and that H19 regulates let-7 (Kallen *et al*, 2013) which targets *Igf1r* (Zhu *et al*, 2011), we wished to determine the physiological role of H19 in the endometrium and its relationship with the IGF signaling in endometriosis.

In this report, we show that the expression of both *H19* and *Igf1r* is significantly decreased in the eutopic endometrium of women with endometriosis. We provide evidence that decreased *H19* expression leads to increased bioavailability of let-7, which in turn inhibits *Igf1r* expression at the post-transcriptional level, thereby contributing to reduced stromal cell proliferation. Perturbation of this newly identified H19/Let-7/IGF1R regulatory pathway may contribute to impaired endometrial preparation and receptivity in women with endometriosis.

## Results

### The expression of *H19* and *Igf1r* is decreased in the endometrium of women with endometriosis

**Figure 1:**
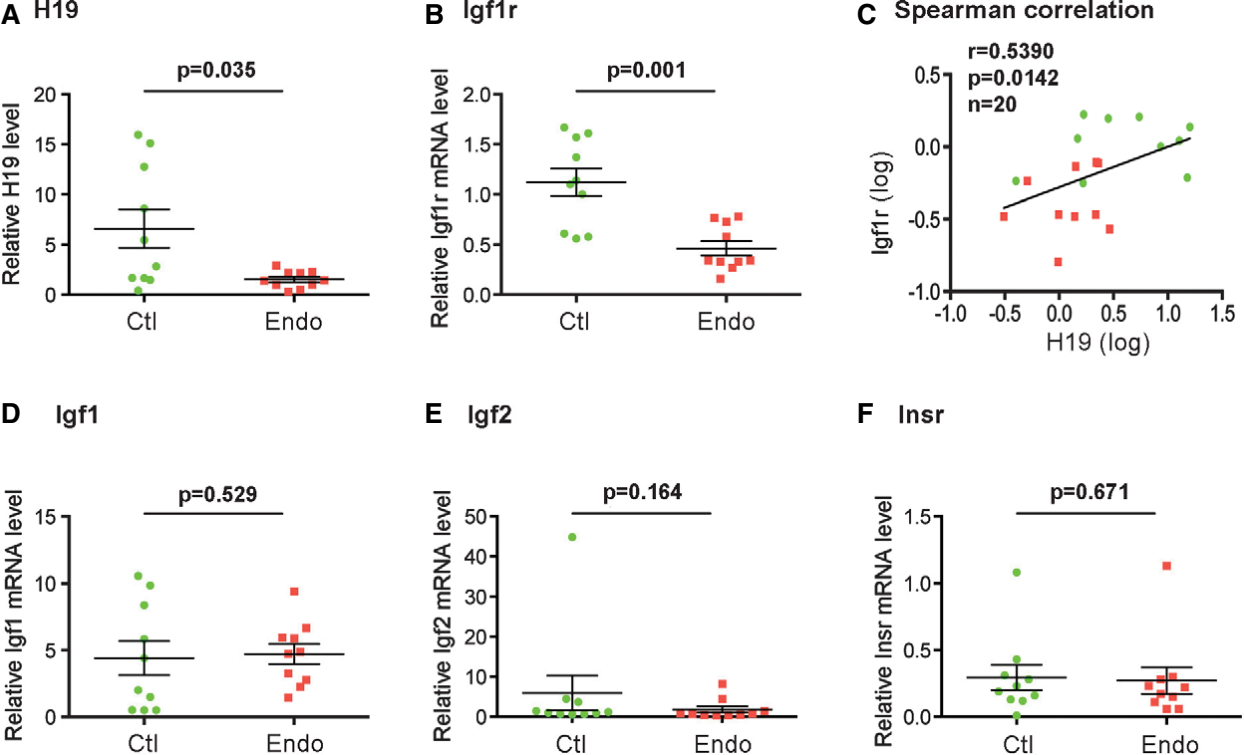
The expressions of *H19* and *Igf1r* are significantly decreased in the eutopic endometrium of women with endometriosis compared to those without endometriosis A–F (A, B, D–F) RNA levels determined by RT–qPCR. Results are presented as mean ± SD,*n* = 10. *P*-values are indicated on the top of each plot. (C) Spearman correlation suggests an *in vivo* positive correlation between the expressions of *H19* and *Igf1r* in a statistically significant manner. Spearman correlation coefficient, *P*-values, and sample numbers are marked on the top left of the plot.

To determine whether the expressions of *H19*, *Igf1r*, *Igf1*, *Igf2*, and *Insr* might be altered in the endometrium of woman with endometriosis, eutopic endometrial biopsies were collected during the late proliferative phase from women with and without endometriosis (see Appendix Table S1 for characterization of the patients). The RNA levels were assessed by reverse-transcription and quantitative real-time PCR (RT–qPCR). Mann–Whitney’s *U*-test revealed a significant H19 decrease in the endometriosis (Endo) compared to the nonendometriosis (Ctl) group (medians of Endo and Ctl were 1.96 and 8.10, respectively; *P* = 0.035, Fig1A). Intriguingly, there was a concomitant decrease in Igf1r in the Endo compared to the Ctl group (medians of Endo and Ctl were 0.46 and 1.11, respectively; *P* = 0.001, Fig1B). Spearman correlation suggested a positive relationship between H19 and Igf1r (Fig1C, *P* = 0.014), implicating possible *in vivo* interactions between the two genes. On the contrary, there was no significant difference in the levels of Igf1, Igf2, and Insr between the two groups (Fig1D–F). Together, these results suggested that decreased expression of *H19* and *Igf1r* might contribute to impaired development and function of eutopic endometrium in patients with endometriosis.

### The H19/Let-7 axis regulates *Igf1r* expression in endometrial stromal cells

**Figure 2:**
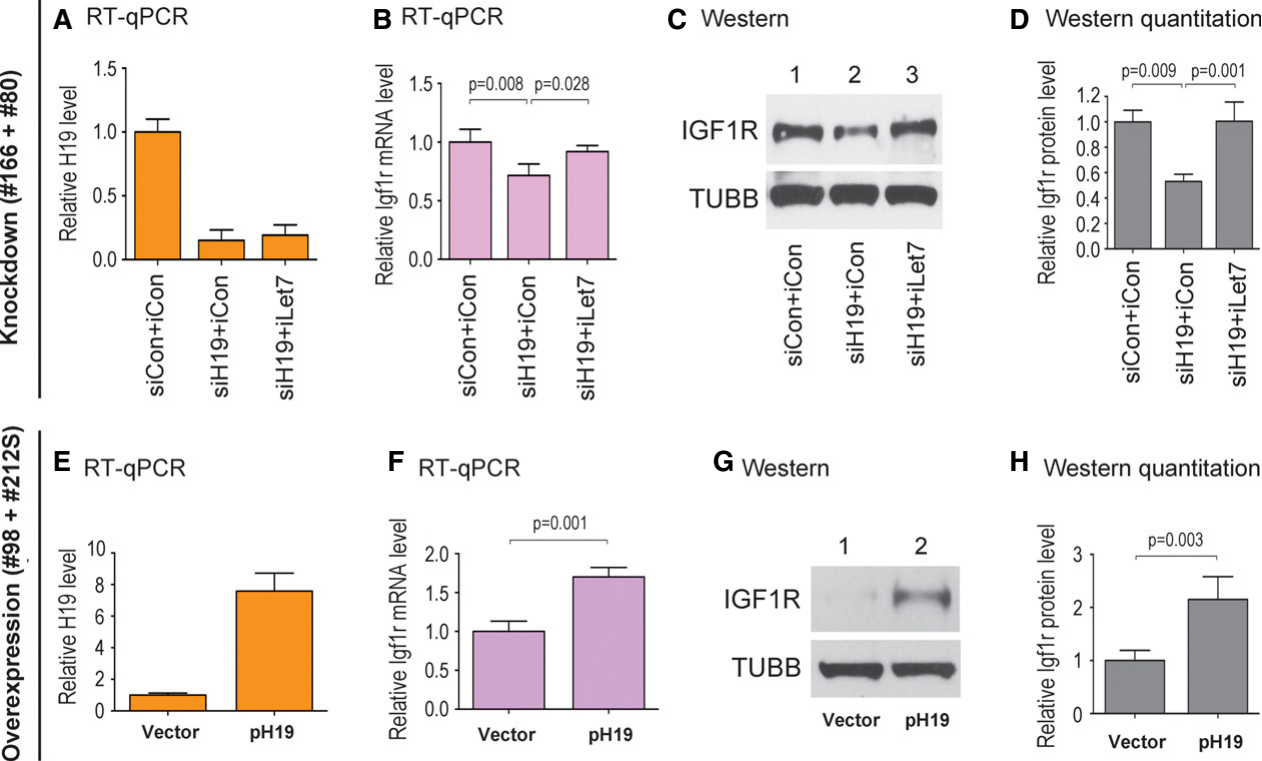
H19 promotes *Igf1r* expression in endometrial stromal cells by inhibiting let-7 A–D Endometrial stromal cells from patients #166 and #80 were each transfected with a mixture of siCon (control siRNA) and microRNA inhibitor control (iCon), siH19 and iCon, or siH19 and iLet7 (let-7-specific inhibitor). RNA and proteins were analyzed at 48 h post-transfection. Combined results from the two patient cells are presented. Western blot gels from #166 cells are shown in (C). Quantitation of Western blots combining both patient cells are shown in (D).

**Figure 3:**
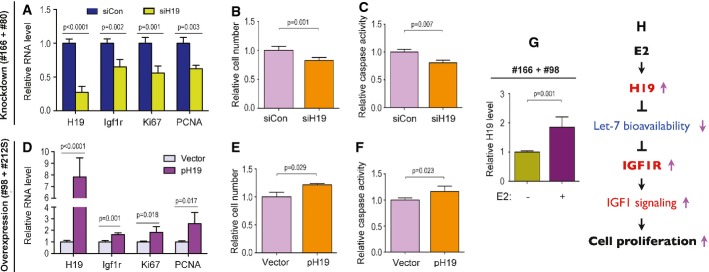
H19 stimulates proliferation of endometrial stromal cells A–F The indicated cells were transfected with siCon, siH19, empty vector, or pH19. RNA, cell viability (as indicated by viable cell numbers), and caspase activity (as a readout for apoptosis) were analyzed 48 h post-transfection. Combined results from two patient cells in each group are presented.

To begin to elucidate the molecular pathway by which H19/Igf1r might regulate endometrial cell function, we used primary endometrial stromal cells derived from human endometrial biopsies as a model, as these cells express both H19 and Igf1r *in vivo* (Tang *et al*, 1994; Ariel *et al*, 1997; Tanos *et al*, 2004). We previously demonstrated that in skeletal muscle and tumor cells H19 functions to reduce the bioavailability of let-7 by acting as a molecular sponge (Gao *et al*, 2014; Yan *et al*, 2014). H19 contains multiple let-7-binding sites that sequester let-7 and prevent it from binding to its target mRNA (Kallen *et al*, 2013). Thus, it is the bioavailability of let-7, hence the relative expression levels between H19 and let-7 and not its absolute expression level, that determines target gene expression. Both human and mouse Igf1r mRNA contain let-7-binding sites at its 3′ UTR, and Igf1r is a validated target of let-7 (Zhu *et al*, 2011). Given the concomitant downregulation of *H19* and *Igf1r* in the eutopic endometrium of endometriosis (Fig1A and B), we sought to determine whether H19, through sequestering let-7, might regulate *Igf1r* expression in the human endometrial stromal cells. Thus, H19 knockdown and overexpression experiments were performed in endometrial stromal cells derived from four patients with endometriosis. We performed knockdown experiments in two patient cells (#166 and #80) that expressed high levels of endogenous H19, and overexpression experiments in other two patient cells (#98 and #212S) with relatively low endogenous H19 (Appendix Fig S1). The rationale for selecting low H19 cells for overexpression was to avoid possible overexpression-induced artifact. Combined results from two patient cells in each group are presented in Figs2 and 3, with results of each individual cells shown in Appendix Figs S2 and S3.

E–H Endometrial stromal cells from patients #98 and #212S were each transfected with empty vector or pH19. RNA and proteins were analyzed 48 h post-transfection. Combined results from the two patient cells are presented. Western blot gels from #98 cells are shown in (G). Quantitation of Western blots combining both patient cells are shown in (H). Data information: Results are presented as mean ± SD, *n* = 6. *P*-values are indicated on the top of each plot.

G E2 stimulates *H19* expression in endometrial stromal cells. Endometrial stromal cells #166 and #98 were stimulated with E2 (+) or vehicle (−) for 48 h, followed by RT–qPCR analysis of RNA extracted from the cells. Results combining two patient cells are shown.

H A proposed model of the H19/let-7/IGF1R-mediated regulation of endometrial stromal cell proliferation. During the proliferative phase of the endometrium, E2 stimulates the expression of *H19*. As its level rises, H19 acts as a sponge to sequester let-7 and prevent it from inhibiting its target gene *Igf1r*. Increased IGF1R protein level leads to increased IGF1 signaling with a biological endpoint of increased proliferation of endometrial stromal cells. Data information: Results are presented as mean ± SD, *n* = 6. *P*-values are indicated on the top of each plot.

For H19 knockdown experiments, a siRNA specific for human H19 (siH19, Kallen *et al*, 2013; Yan *et al*, 2014) was used in the presence and absence of let-7-specific inhibitor (iLet7, Kallen *et al*, 2013; Yan *et al*, 2014), followed by the analysis of *Igf1r* expression at 48 h post-transfection. iLet7s are chemically modified, single-stranded nucleic acids that specifically bind to let-7 and block its function. The purpose for using iLet7 was to confirm the contribution of let-7 to the H19-mediated pathway, as H19 has other functions in addition to sequestering let-7 (Keniry *et al*, 2012; Venkatraman *et al*, 2013; Dey *et al*, 2014; Monnier *et al*, 2014). The effect of H19 knockdown (i.e., downregulation of *Igf1r*) would be abrogated in the presence of iLet7, which acts to neutralize let-7 released from H19 sequestration. When H19 was downregulated by siH19 in the absence of iLet7 (Fig2A, compare the middle column to the left column), there was a decrease in the level of Igf1r mRNA (Fig2B, compare the middle column to the left column). This suggested that H19 deficiency led to enhanced let-7 action, enabling it to repress the expression of *Igf1r*, a known target of let-7 (Zhu *et al*, 2011). The level of Igf1r mRNA was restored to that of control (Fig2B, compare the right column to the left column) when H19 was knocked down in the presence of iLet7 (Fig2A, compare the right column to the left column). Changes in the Igf1r protein level in response to H19 knockdown with or without iLet7 closely mimicked those of the Igf1r mRNA (Fig2C and D). Results from the individual patient cells are shown in Appendix Fig S2A–H. Together, these results suggested that in endometrial stromal cells H19 regulates *Igf1r* expression post-transcriptionally by affecting the bioavailability of let-7. As the expression of *Igf1r* could be fully rescued through blocking let-7 by iLet7 in the context of H19 knockdown at both mRNA and protein levels (Fig2A–D), the contribution of let-7 in the H19-mediated pathway in endometrial stromal cells is strongly supported.

To provide further evidence in support of this notion, H19 overexpression experiments were carried out. Thus, the cells were transfected with a human H19-expressing vector pH19 (Kallen *et al*, 2013; Yan *et al*, 2014) or empty vector, and effects on *Igf1r* expression were analyzed 48 h later. When H19 was overexpressed (Fig2E, compare right column with the left column), an increase in the Igf1r mRNA level was observed (Fig2F, compare right column with the left column). There was an increase in the Igf1r protein level as well (Fig2G and H, compare the right columns with the left column). The lower Igf1r protein level in Fig2G, lane 1, compared to that in Fig2C, lane 1, was consistent with the relatively lower H19 levels in the corresponding cells (Appendix Fig S1). Results from the individual patient cells are shown in Appendix Fig S2I–P. These results were consistent with the view that an elevated level of H19 led to reduced bioavailability of let-7, which in turn relieved the inhibition of *Igf1r* expression by let-7. Combining with the siRNA knockdown results (Fig2A–D), we concluded that H19 regulates *Igf1r* expression at the post-transcriptional level in human endometrial stromal cells and that this regulation is at least in part mediated by let-7.

### H19 promotes proliferation of endometrial stromal cells

It is well established that IGF1 acts on its receptor IGF1R to activate the PI3K/AKT and Ras/Raf/MAPK signal transduction pathways, thereby promoting uterine cell proliferation (Tang *et al*, 1994; LeRoith *et al*, 1995; Ivanga *et al*, 2007). Indeed, *Igf1r* overexpression with heightened AKT activity has been detected in hyperplastic endometrium (McCampbell *et al*, 2006). Our results have demonstrated that in endometrial stromal cells H19 promotes *Igf1r* expression via reducing the bioavailability of let-7 (Fig2; Appendix Fig S2). Based on these findings, we predicted that H19 knockdown or overexpression would negatively or positively affect the proliferation of endometrial stromal cells. Results shown in Fig3; Appendix Fig S3 are consistent with our prediction.

When H19 was knocked down by siH19 (Fig3A, first column on the left, compare blue bar to green bar), the level of Igf1r mRNA was decreased as expected (second column). Under these conditions, a significant decrease in the number of viable cells was also observed (Fig3B) even with decreased cell death (Fig3C). This suggested that downregulation of H19 caused decreased proliferation of endometrial stromal cells, which was further confirmed by decreased expression of cell proliferation markers Ki67 (Scholzen & Gerdes, 2000) and PCNA (Moldovan *et al*, 2007) (Fig3A, right two columns, compare green bars to pink bars). Consistent with the knockdown results, overexpression of H19 (Fig3D, left column) led to an increase in the expression of *Igf1r*, *Ki67*, and *PCNA* (second through fourth columns), with a concomitant increase in the number of viable cells (Fig3E) even in the presence of more apoptotic activity (Fig3F). As H19 knockdown simultaneously reduced cell proliferation and cell death (Fig3B and C) and its overexpression elicited opposite effects (Fig3E and F), a dual role of H19 in regulating endometrial stromal cell growth and survival could be proposed, although the mechanism underlying the putative H19-mediated crosstalk remains to be investigated. Results from the individual patient cells are shown in Appendix Fig S3.

### Estrogen stimulates H19 expression in endometrial stromal cells

In the mouse uterus, *H19* expression is stimulated by 17-beta estradiol (E2) and follows a cyclic pattern (Adriaenssens *et al*, 1999). The expression is lowest during the proestrus and gradually increases during the proliferative phase until ovulation. It remains high during the early secretory phase and sharply declines during the late secretory phase. A similar cyclic pattern of *H19* expression was observed in the human endometrium (Ariel *et al*, 1997). Interestingly, the expression pattern of *Igf1r* in the human endometrium mimics that of *H19* (Ivanga *et al*, 2007). Given that E2 acts as a potent stimulator of IGF1 signaling in the endometrium (Ivanga *et al*, 2007) and that H19 stimulates *Igf1r* expression (Fig2; Appendix Fig S2), we hypothesized that E2 may stimulate *H19* expression which in turn upregulates *Igf1r* expression, thereby promoting IGF1 signaling in the human endometrial cells. To test this hypothesis, human endometrial stromal cells were treated with E2 and *H19* expression was analyzed after 48 h of treatment. Results showed that E2 stimulated *H19* expression by approximately 2-fold (Fig3G) irrespective of endogenous H19 levels (Appendix Fig S2), which was consistent with our hypothesis.

## Discussion

This study investigates the long noncoding RNA H19, its interaction with the IGF signaling pathway via the microRNA let-7, and the role they play in the human endometrium. Women with endometriosis have altered gene expression in the eutopic endometrium that may impair fertility. Our results show that women with endometriosis have decreased *H19* expression as well as a concomitant decrease in the level of Igf1r mRNA in the eutopic endometrium compared to women without endometriosis. Using primary cultured human endometrial stromal cells as a model, we provide evidence that H19 regulates *Igf1r* expression in endometrial stromal cells by sequestering let-7 and reducing its bioavailability. We show that H19 promotes proliferation of endometrial stromal cells and, furthermore, estradiol stimulates *H19* expression, which upregulates *Igf1r* and promotes IGF1 signaling. The combination of results from our *in vivo* and *in vitro* studies strongly suggests a regulation of *Igf1r* by H19 and its physiological relevance in the human endometrium. However, it is almost certain that additional regulatory circuits are involved in the H19/Igf1r pathway given that H19 is a multi-functional RNA and that *Igf1r* can be regulated by mechanisms other than H19.

We did not observe a statistically significant difference in *H19* expression in the cultured endometrial stromal cells from women with and without endometriosis (data not shown). This was inconsistent with our *in vivo* data demonstrating significantly lower *H19* expression in women with endometriosis as compared to woman without endometriosis (Fig1A). This apparent discrepancy is likely the result of *in vitro* manipulation of isolated stromal cells. The stress of *in vitro* culture combined with the loss of *in vivo* crosstalk with other cell types may have contributed to the altered *H19* expression *in vitro*, making it less reflective of the *in vivo* condition. In support of this notion, *in vitro* culture of human placental mesenchymal stem cells has been reported to induce epigenetic alterations (Zhu *et al*, 2015). Despite the above-mentioned caveat, the combined results from four different patient cells, with both H19 knockdown and overexpression, strongly point to the notion that the H19/Let-7/IGF1R pathway is likely operating in controlling endometrial stromal cell proliferation. Thus, primary endometrial stromal cells remain a valuable *in vitro* model for the mechanistic dissection of the molecular interplay between H19 and its regulated genes, with the potential of identifying novel therapeutic targets for future intervention.

Endometriosis has been traditionally viewed as a proliferative disorder with growth of endometrial cells in ectopic locations; however, previous data have shown proliferation defects in the eutopic endometrium of women with endometriosis compared to women without endometriosis. Women with endometriosis develop significantly lower peak endometrial thickness when compared to women without endometriosis (Bromer *et al*, 2009). Normal eutopic endometrium grows quickly during the proliferative phase of the menstrual cycle, but our findings suggest that alterations of the H19/Let-7/IGF1R pathway may impair eutopic endometrial cell growth in women with endometriosis.

It has been proposed that endometriosis causes adhesions and scarring of the pelvic anatomy, which can impair oocyte release or cause tubal blockage (Schenken *et al*, 1984). Additionally, patients may have altered peritoneal function with an increase in inflammatory cytokines due to peritoneal disease or involvement. Two large randomized controlled trials which compared the fecundity of surgical treated and untreated women with endometriosis concluded that women with surgically treated mild endometriosis continue to have impaired fecundity, suggesting that visible endometriotic lesions do not represent the sole mechanism of the fertility impairment (Marcoux *et al*, 1997; Parazzini, 1999). Indeed, a number of studies have suggested that endometriosis causes dysfunction of the eutopic endometrium. Women with endometriosis display aberrant expression of genes in their eutopic endometrium, many of which (including HOXA10, aromatase, progesterone receptors, matrix metalloproteinases, and ab-integrin) have been implicated in endometrial development and receptivity (reviewed in Macer & Taylor, 2012; Hurst *et al*, 2014). Our results unveil new gene regulatory pathways that may underlie the dysfunctional endometrial development seen in women with endometriosis.

In light of our findings described in this manuscript, we propose the following model of the H19/Let-7/IGF1R-mediated regulation of endometrial cell proliferation (Fig3H). During the proliferative phase of the menstrual cycle, estradiol stimulates the expression of *H19* in the endometrium. As levels of H19 rise, it acts as a sponge to sequester let-7, thereby preventing let-7 from inhibiting its target gene *Igf1r*. Increased Igf1r protein levels lead to increased IGF1 signaling with a biological endpoint of increased proliferation of endometrial stromal cells. This pathway appears to be altered in women with endometriosis. Endometriosis patients have decreased *H19* expression at the level of eutopic endometrium, which leads to increased let-7 bioavailability. This allows for the increased inhibition of *Igf1r* and thus decreased IGF1 signaling, which ultimately leads to decreased stromal cell proliferation. Alterations in this H19/Let-7/IGF1R-mediated regulation of endometrial cell proliferation may represent a potential mechanism for infertility in women with endometriosis. Further research is needed to improve our understanding of this pathway and to identify novel molecular targets for intervention and treatment of infertility in this patient population.

## Materials and Methods

### Materials

Antibodies for IGF1R (Cell Signaling, 3027; used at a dilution of 1:1,000) and β-tubulin (TUBB) (Abcam, ab6046; used at a dilution of 1:3,000) were purchased. Human H19-specific siRNA (siH19), nontargeting siRNA (siCon), let-7 inhibitor (iLet7), anti-miR control (iCon), plasmids expressing human H19 (pH19), and empty vector were previously described (Kallen *et al*, 2013; Yan *et al*, 2014). 17β-estradiol (E2) was purchased from Sigma-Aldrich (E8875-1G).

### Tissue sample collection

Women undergoing laparoscopic surgery for suspected endometriosis or idiopathic infertility at Bern Hospital in Switzerland were identified. Informed consent was obtained for each patient prior to surgery. Surgery was scheduled to occur during the late proliferative phase of the menstrual cycle, and endometrial tissue samples were collected at the time of surgery. For all the patients in the study, samples of eutopic endometrium were collected using a soft curette (Pipelle-de-Cornier, Laboratoire CCD, France). For patients with confirmed endometriosis, a sample of ectopic endometrium was also collected. After the samples were collected, the tissue was stored in RNAlater at −80°C. Endometriotic lesions were confirmed by histological examination. Patient data were also collected including the use of hormonal medication in the 3 months prior to surgery, age, and body mass index (BMI). The tissue collected for this study included eutopic endometrial biopsies from 10 women with endometriosis and 10 women without endometriosis.

### Analysis of gene expression in eutopic endometrial tissue

For eutopic endometrial biopsies, approximately 30 mg of tissue was excised and homogenized in a FastPrep 120 tissue homogenizer (30 s at 4.0 m/s) in cell lysis buffer (Qiagen, Hombrechtikon, Switzerland). The remaining RNA isolation was performed with the RNAeasy mini kit (Qiagen) and TurboDNase (Ambion, Life Technologies, Zug, Switzerland) for genomic DNase digestion. One microgram of total RNA was reverse-transcribed in a 25-μl reaction with the Moloney murine leukemia virus (MMLV) reverse transcriptase (Promega, Dübendorf, Switzerland) and random primers. The resulting cDNA was diluted 1:20, and the absence of genomic DNA was confirmed with a reverse-transcriptase negative control.

The quantitative real-time polymerase chain reaction (qPCR) was performed with the Rotor-Gene FAST SYBR green PCR kit (Qiagen) in a Rotor-Gene RG 2000 (Corbett Research, NSW, Australia) under the following conditions: 95°C for 5 min, followed by 40 cycles of 95°C for 5 s and 60°C for 10 s. Primers were synthesized by Microsynth, Balgach, Switzerland, and the sequences are defined in Appendix Table S2. Specificity of amplification was confirmed by melting curve analysis and the absence of amplification in the no-template control.

### Statistical analysis of data from human tissue samples

The most appropriate reference genes for the tissue included in this study were determined from a panel of 8 reference genes and using the geNORM program as part of the qBASE+ software suite (Biogazelle, Zwijnaarde, Belgium) (Vandesompele *et al*, 2002). Reaction efficiency of each gene was also included in the analysis and was calculated via linear regression (Ruijter *et al*, 2009). Analysis of the reference gene panel indicated that a panel of at least seven reference genes was required to reach an adequately low variability (< 0.15) among the samples in the study.

Gene expression was determined based on the ΔΔ*C*_t_ method and calculated by the qBASE+ software. Results are expressed as fold change from a standard reference sample included in each run. The analyses of gene expression were calculated via a nonparametric Mann–Whitney *U*-test. *P*-values of 0.05 or less were considered significant.

### Human endometrial stromal cell isolation and culture

Endometrial tissues were obtained from healthy reproductive aged women undergoing elective gynecological surgery or women diagnosed with endometriosis. Tissue collection and usage were approved by the Yale Human Investigations Committee. Endometrial stromal cells were isolated using the methods previously reported (Yang *et al*, 2012). Cells were maintained in DEME/F12 (1:1) (Gibco, Life Technologies, 11330-032), supplemented with 10% FBS, 1 mM L-glutamine, and 1% penicillin–streptomycin in 5% CO_2_ atmosphere at 37°C.

### RT–qPCR and Western blot analyses

RT–qPCR and Western blot analysis were carried out as previously described Kallen *et al* (2013). The PCR primers for the indicated human genes are listed in Appendix Table S2.

### H19 siRNA knockdown and let-7 inhibitor rescue and H19 overexpression experiments

Cells were transfected in a 24-well plate scale. To prepare siRNA transfection solution for each well, 30 pmol of siCon or siH19 was mixed with 100 μl OPTI-MEM by gentle pipetting. In parallel, 3 μl Lipofectamine 2000 was mixed with 100 μl OPTI-MEM. Following 5 min of incubation at room temperature (RT), the two were mixed by gentle pipetting and incubated for 20 to 30 min at RT to allow siRNA/lipid complexes to form. At the end of incubation, the 200 μl transfection solution was used to re-suspend the cell pellet (∼1.5 × 10^5^ cells/well). After incubation at RT for 10 min, regular growth medium was added at a ratio of 1:5 (1 volume of transfection solution/4 volumes of growth medium) and the cell suspension was transferred to the culture plate. After 24-h incubation at 37°C in 5% CO_2_, the medium was replaced with fresh growth medium. RNAs and proteins were extracted and analyzed at the indicated time points following transfection.

Plasmid DNA transfections were carried out as described for siRNA, except that 1 μg DNA in 25 μl OPTI-MEM and 1 μl Lipofectamine 2000 in 25 μl OPTI-MEM were used for each well of cells (the 50 μl of final transfection reagent with 500 μl of regular growth medium was added to each well). For iLet7 rescue experiments, 30 pmol of siCon/siH19 and 60 pmol of iCon/iLet7 were used for each well of cells.

### Cell viability and apoptosis

These were performed as previously described Yan *et al* (2015). Briefly, cells were transfected and seeded in 96-well plates at a density of 5 × 10^3^/well. Cell viability and caspase 3/7 activity were measured 48 h post-transfection using the CellTiter-Blue Cell Viability kit (Promega) and the Apo-ONE Homogeneous Caspase-3/7 Assay kit (Promega), respectively, according to the manufacturer’s protocols.

### E2 treatment of human endometrial stromal cells

Cells were seeded in 24-well plates in regular complete growth media at a density of ∼1.5 × 10^5^ per well. The next day, media were replaced with serum-free media containing E2 (final concentration of 10^−8 ^M), followed by incubation for the indicated time duration. E2 was replenished every 24 h in fresh serum-free media until RNA extraction for further analyses.

### Statistical analysis of data from human stromal cell experiments

All data are presented as mean ± standard deviation (SD) unless otherwise indicated. Data were analyzed using two-tailed Student’s *t*-test for the average differences. Statistical analyses were performed using the Statistical Package for the Social Science (SPSS) computer software version 17.0 (IBM SPSS Statistics, Chicago, IL, USA). Figures were constructed using Prism 6 version 6.0f (GraphPad Software, Inc.). *P*-values of 0.05 or less were considered significant.

## Supporting Information

**Appendix**

**Review Process File**
