# Supplementary material for: H19 lncRNA alters stromal cell growth via IGF signaling in the endometrium of women with endometriosis
Source: EMBO Mol Med. 2015 Jun 18;7(8):996–1003. doi: 10.15252/emmm.201505245 (PMC4551339; doi:10.15252/emmm.201505245)
Supplement: Supplementary file 1 [file emmm0007-0996-sd1.pdf]

**Appendix PDF**

**Table of Contents:**

1. Appendix Table S1
2. Appendix Table S2
3. Appendix Figure S1
4. Appendix Figure S2
5. Appendix Figure S3

**Appendix Table S1.** Clinical data of patient samples with *in vivo* H19 and Igf1r expression levels shown

| | | Patient Code | Age | BMI | Hormonal treatment | *H19* | *Igf1r* |
|---|---|---|---|---|---|---|---|
| ***Ctl*** ***(Non-endometriosis)*** | 1 | **Eu413** | 27 | 23.0 | No | 3 | 2.14 |
| | 2 | **Eu507** | 19 | 19.1 | No | 9.04 | 2.35 |
| | 3 | **Eu530** | 28 | 25.9 | No | 13.06 | 1.21 |
| | 4 | **Eu531** | 24 | 19.1 | No | 18.89 | 0.54 |
| | 5 | **Eu548** | 19 | 22.4 | No | 18.55 | 1.51 |
| | 6 | **Eu555** | 33 | 23.2 | No | 7.16 | 2.82 |
| | 7 | **Eu559** | 35 | 26.6 | No | 2.33 | 0.66 |
| | 8 | **Eu603** | 35 | 18.9 | No | 1.83 | 1 |
| | 9 | **Eu473** | 35 | 22.0 | No | 10.93 | 0.41 |
| | 10 | **Eu634** | 35 | 26.9 | No | 1.6 | 0.58 |
| | | **Mean ± SEM** | 29.00 ± 2.08 | 22.71 ± 0.97 | | 8.64 ± 2.10 | 1.32 ± 0.27 |
| ***Endo*** ***(Endometriosis)*** | 1 | **Eu20** | 30 | 21.9 | No | 3.31 | 0.52 |
| | 2 | **Eu57** | 28 | 20.2 | No | 2.94 | 0.4 |
| | 3 | **Eu139** | 26 | 33.1 | No | 1.72 | 1.07 |
| | 4 | **Eu417** | 28 | 20.1 | No | 0.91 | 0.35 |
| | 5 | **Eu452** | 26 | 28.4 | No | 2 | 0.38 |
| | 6 | **Eu541** | 24 | 16.3 | No | 3.27 | 1.36 |
| | 7 | **Eu674** | 30 | 23.2 | No | 4.16 | 1.91 |
| | 8 | **Eu209** | 27 | 28.7 | No | 1.86 | 0.2 |
| | 9 | **Eu463** | 22 | 25.5 | No | 1.06 | 1.14 |
| | 10 | **Eu604** | 31 | 23.8 | No | 1.93 | 0.38 |
| | | **Mean ± SEM** | 27.20 ± 0.89 | 24.12 ± 1.57 | | 2.31 ± 0.33 | 0.77 ± 0.18 |

**Appendix Table S2.** Real-time PCR primers

| Gene | Forward primer | Reverse primer |
|---|---|---|
| β Tubulin | 5′-CGTGTTCGGCCAGAGTGGTGC | 5′-GGGTGAGGGCATGACGCTGAA |
| Gapdh | 5’-TGCACCACCAACTGCTTAGC | 5’-GGCATGGACTGTGGTCATGAG |
| Actin β | 5’-CTGGAACGGTGAAGGTGACA | 5’-AAGGGACTTCCTGTAACAATGCA |
| YWHAZ | 5’-ACTTTTGGTACATTGTGGCTTCAA | 5’-CGCCAGGACAAACCAGTAT |
| RPL13A | 5’-CCTGGAGGAGAAGAGGAAAGAGA | 5’-TTGAGGACCTCTGTGTATTTGTCAA |
| SDHA | 5'-TGGGAACAAGAGGGCATCTG | 5'-CCACCACTGCATCAAATTCATG |
| HPRT1 | 5'-TGACACTGGCAAAACAATGCA | 5'-GGTCCTTTTCACCAGCAAGCT |
| β2M | 5'-TGCTGTCTCCATGTTTGATGTATCT | 5'-GGCATGGACTGTGGTCATGAG |
| H19 | 5′-ACTCAGGAATCGGCTCTGGAA | 5′-CTGCTGTTCCGATGGTGTCTT |
| Igf1r | 5'-GCACCATCTTCAAGGGCAATTTG | 5'-AGGAAGGACAAGGAGACCAAGG |
| Igf1 | 5'-GCGTTTTCCCCCAATGAAAT | 5'-GGTGCAAATCACTCCTAAAGACAA |
| Igf2 | 5'-CCGAAACAGGCTACTCTCCT | 5’-AGGGTGTTTAAAGCCAATCG |
| Insr | 5’-CAGCTGAGCTAGAAGCCAAC | 5'-TGAGTGATGGTGAGGTTGTG |
| Ki67 | 5'-TTGGAGAATGACTCGTGAGC | 5'-CGAAGCTTTCAATGACAGGA |
| PCNA | 5'-CCTTGGCGCTAGTATTTGAA | 5'-TTTCCAAGTTCTCCACTTGC |

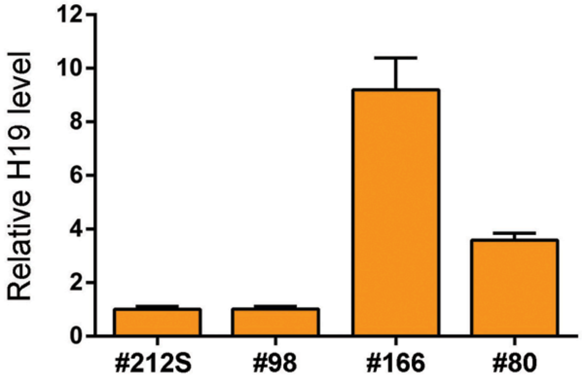


**Appendix Figure S1. H19 is expressed in primary human endometrial stromal cells.** RNAs were isolated from primarily cultured endometrial stromal cells derived from eutopic endometrial biopsies. Relative H19 levels as assessed by RT-qPCR from four patients with endometriosis are shown, with the level of #212S arbitrarily set as 1. #212S, #98, #166, and #80 are patient ID numbers.

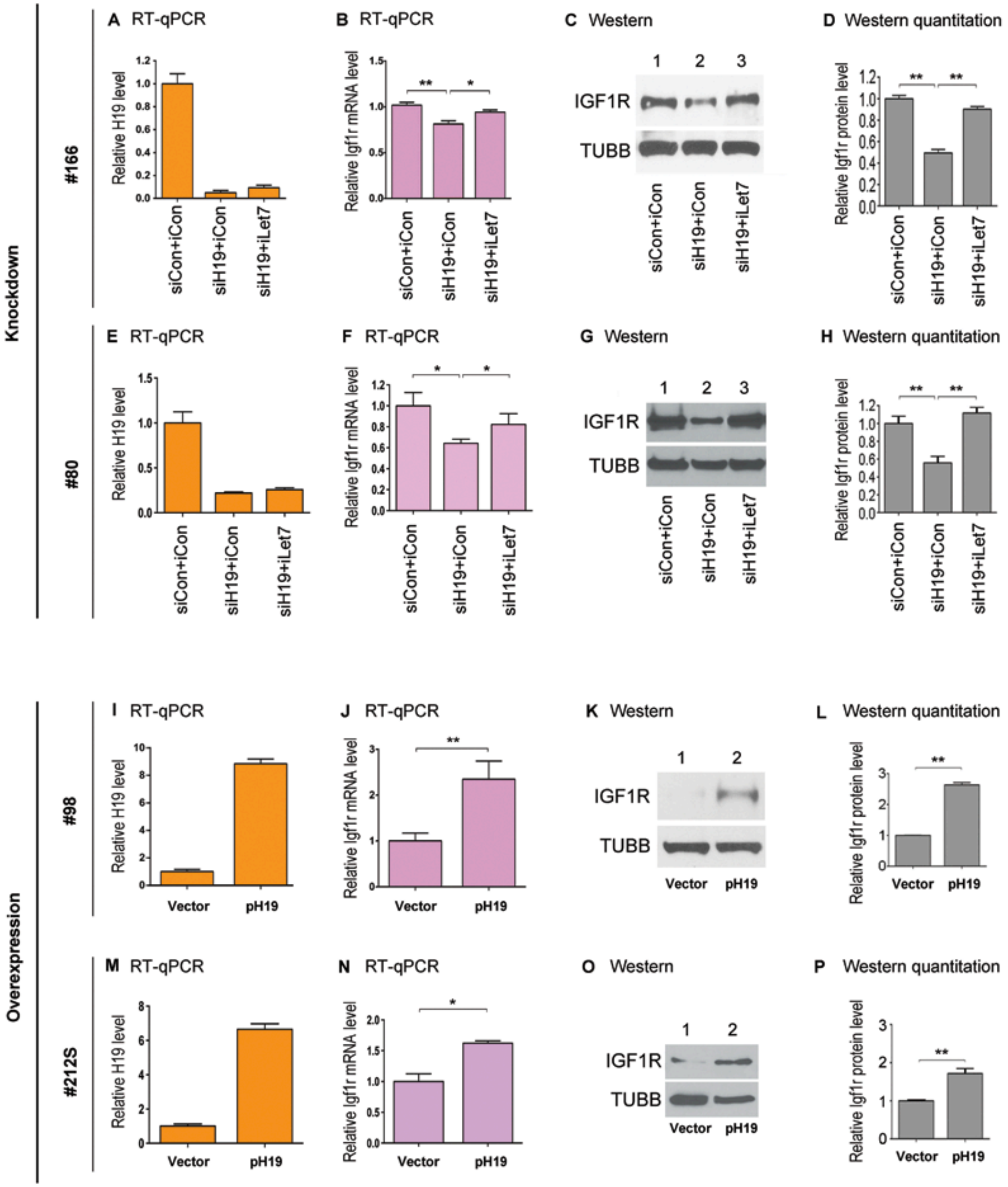


**Appendix Figure S2. Results from four individual patient cells, related to Figure 2.** (**A-H**) Endometrial stromal cells #166 and #80 were each transfected with the indicated mixture. (**I-P**) Endometrial stromal cells #98 and #212S were each transfected with vector or pH19. Results from three independent transfection experiments with each of the four cells are shown. Numbers are mean ± SD (n=3). *, $p < 0.05$; **, $p < 0.01$.

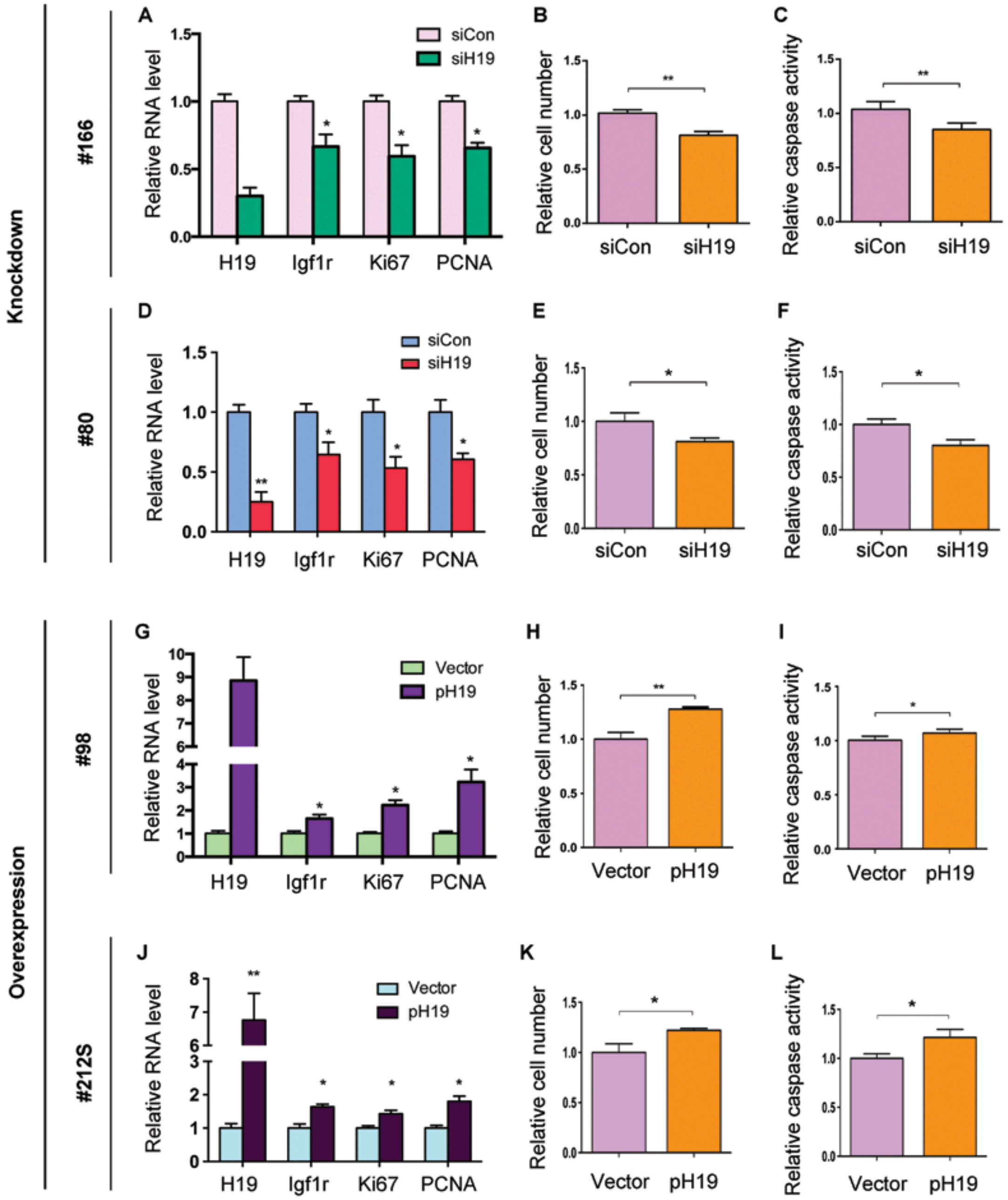


**Appendix Figure S3. Results from four individual patient cells, related to Figure 3.** (**A-F**) Endometrial stromal cells #166 and #80 were each transfected with the indicated mixture. (**G-L**) Endometrial stromal cells #98 and #212S were each transfected with vector or pH19. Results from three independent transfection experiments with each of the four cells are shown. Numbers are mean ± SD (n=3). *, $p < 0.05$; **, $p < 0.01$.
