# Supplementary material for: H19 lncRNA alters stromal cell growth via IGF signaling in the endometrium of women with endometriosis
Source: EMBO Mol Med. 2015 Jun 18;7(8):996–1003. doi: 10.15252/emmm.201505245 (PMC4551339; doi:10.15252/emmm.201505245)
Supplement: Supplementary file 2 [file emmm0007-0996-sd2.pdf]

H19 lncRNA alters stromal cell growth via IGF signaling in the endometrium of women with endometriosis

Sanaz Ghazal, Brett McKinnon, Jichun Zhou, Martin Mueller, Yi Men, Lihua Yang, Michael Mueller, Clare Flannery, Yingqun Huang, Hugh Taylor

Corresponding author: Yingqun Huang, Yale University School of Medicine

Review timeline:

Submission date:	11 March 2015
Editorial Decision:	01 April 2015
Revision received:	07 May 2015
Editorial Decision:	26 May 2015
Revision received:	27 May 2015
Accepted:	28 May 2015

Transaction Report:

(Note: With the exception of the correction of typographical or spelling errors that could be a source of ambiguity, letters and reports are not edited. The original formatting of letters and referee reports may not be reflected in this compilation.)

Editor: Céline Carret

1st Editorial Decision 01 April 2015

Thank you for the submission of your manuscript to EMBO Molecular Medicine. We have now heard back from the two referees whom we asked to evaluate your manuscript. Although the referees find the study to be of interest, they also raise concerns that must be addressed in the next final version of your article.

As you will see, both referees suggest to increase the conclusiveness of the findings by performing additional controls and experiments. Referee 1 also proposes few changes in the way the paper is organised and figures displayed, while referee 2 would require that all replicates are shown and different statistical analysis maybe performed. We would like to particularly draw your attention on the last point and as a related matter, we now require an authors' checklist to be submitted with revised articles.

Therefore, given these evaluations, I would like to give you the opportunity to revise your manuscript, with the understanding that the referee concerns must be fully addressed and that acceptance of the manuscript may entail a second round of review.

EMBO Molecular Medicine has a "scooping protection" policy, whereby similar findings that are published by others during review or revision are not a criterion for rejection. Should you decide to submit a revised version, I do ask that you get in touch after three months if you have not completed it, to update us on the status.

Please also contact us as soon as possible if similar work is published elsewhere. If other work is published we may not be able to extend the revision period beyond three months.

I look forward to receiving your revised manuscript.

***** Reviewer's comments *****

Referee #1 (Comments on Novelty/Model System):

The value of the model system is discussed below in my comments to the author.

Referee #1 (Remarks):

Ghazal et al. describe an interesting set of experiments that uncover a potential role for the H19 lncRNA in endometriosis. The results are of high medical significance and are also quite important in regards to basic science regarding the regulating activity of the insulin growth factors (insulin, insulin-like growth factor 1, insulin-like growth factor 2).

The experimental plan is intelligent and the manuscript is well written.

I do have some suggestions in regards to data presentation and also I think an additional control is required for the experiments described in Figure 2. The paper does provide good evidence for regulation of Igf1 levels via H19+let7 but I think that the authors could be clearer in their analysis and in the Figure 2 experiments to better elucidate the relative importance of let7-dependent and let7-independent pathways.

1. The experiments described in Figure 1 are convincing in regards to demonstrating association of endometriosis with elevated levels of H19 and of IgfR1 (panels A and B). And this demonstration is certainly the main point of Figure 1. However, I am confused by the presentation of the data in panel C. First, it would help greatly to keep the green and red color scheme for the data points. I am also puzzled by the switch to the log scale and wonder about the reasoning for that decision. Finally, I suggest that the authors include (in Supplemental Materials) a table with the values for H19 and Igf1r for each sample. I also think they might want to look at the Control and Endo samples separately. The Endo samples are low for both and may be confounding a fair search for correlation. It actually looks to me that in control data points, high H19 does correlate with relatively high Igf1r but that high Igf1r does not predict much about the H19 levels suggesting multiple forms of regulation in controls. Overall, the R value is not very high, probably because multiple things are going on. I think it would make a stronger paper to address this more clearly.

2. It looks like the data in Figure S1 are normalized to H19 levels in cell line 168. Is that correct? I think it will be much more informative to normalize data the same way as in Figure 1 so that the reader can compare the levels of H19 in these cells relative to that seen in the patient and control samples as described in Figure 1. Right now, I have no idea whether the levels of gene expression in the cell lines are similar to those in vivo or are 100-fold higher or lower, for example.

3. Are cell lines 168, 98, 166, and 80 derived from patients from the experiments in Figure 1? If so, can the values for the H19 levels in tissue be included in Figure S1 so that I can assess for myself how well the in vivo and in vitro results correlate?

4. The authors described several caveats associated with the use of the in vitro system but in the end they make a strong and convincing case for using cell culture models to addressing molecular mechanisms.

5. The experiments described in Figure 2 (A-D) are missing a important control. To address the critical question of whether H19 is working primarily through let7, the essential control data are actually the levels of expression of H19 and Igf1r in cells treated with siCon + iLet7. That is, the question posed by the authors is whether there is any additional effect of H19 once you take let7

regulation of the picture. This siCon + iLet& control is critical for getting some sense of the relative importance of the H19/let7 interactions.

6. Which cell line was used for the experiments described in Figure 2? Do the authors imagine they might see different results (especially in panels E-H) if they used cell lines with relatively high vs relatively low levels of H19.

7. Would there be value in including direct measures of let7 for the studies described in Figure 2.

The paper is very well written so my editorial comments are minor:

1. On page 10, "These results suggested that an elevated level of H19 led to reduced bioavailabllity of let-7......" The experiments are consistent with this but they do not actually address this question. Maybe you could say "These results are consistent with..."

Referee #2 (Comments on Novelty/Model System):

The experiments in this manuscript use established molecular methods performed with tissues and cells from women with and without endometriosis.

Referee #2 (Remarks):

The manuscript of Ghazal and co-authors detail experiments performed using endometrium gathered from women with and without endometriosis. The use of established molecular techniques and primary cell cultures from tissues of affected individuals increases the translational impact of this research. I do have a few concerns and comments which I feel should be addressed before publication.

Supplemental Table 1: Please include average age/BMI +/-SEM for all treatment groups within the body of the Methods section.

Overall, there is quite a lot of discussion presented in the Methods section (i.e. lines 207-218), which would be better presented in the Discussion to improve clarity of the Results section.

Line 207: Expression changes in H19 from tissue collection to culture should be confirmed by either PCR or in situ hybridization to confirm results presented in Figure 1; otherwise this could be due to increased numbers of individuals within analyses rendering previous data insignificant. There appears to be a wide range of H19 expression within "normal" control women presented in Figure 1A, this may extend into the affected population when increased numbers are examined.

Line 229: You should not "cherry pick" which results to present; otherwise instead of presenting data from three replicates you are presenting one replicate. Normalize data to changes in baseline expression to decrease variation between patients, and consult a statistician to help with the analyses. This is a common practice for data generated from studies in higher species where individual variation is high, unlike those in rodent models where similar genetic background controls variability.

Figure 2C vs 2G: By Western blot, your controls in 2C express IGF1R, but the empty vector control in 2G does not express IGF1R. You should present data from all replicates to convince me that your protein expression results are a result of the H19 knockdown/ over expression.

Point-by-point response to referees' comments (reviewers' comments in italics)

Referee #1

Ghazal et al. describe an interesting set of experiments that uncover a potential role for the H19 lncRNA in endometriosis. The results are of high medical significance and are also quite important in regards to basic science regarding the regulating activity of the insulin growth factors (insulin, insulin-like growth factor 1, insulin-like growth factor 2).

The experimental plan is intelligent and the manuscript is well written.

I do have some suggestions in regards to data presentation and also I think an additional control is required for the experiments described in Figure 2. The paper does provide good evidence for regulation of Igf1 levels via H19+let7 but I think that the authors could be clearer in their analysis and in the Figure 2 experiments to better elucidate the relative importance of let7-dependent and let7-independent pathways.

1. The experiments described in Figure 1 are convincing in regards to demonstrating association of endometriosis with elevated levels of H19 and of IgfR1 (panels A and B). And this demonstration is certainly the main point of Figure 1. However, I am confused by the presentation of the data in panel C. First, it would help greatly to keep the green and red colour scheme for the data points.

Response: Thanks much for the suggestion. We have modified Fig. 1C to keep the colour scheme: green for Ctl and red for Endo.

I am also puzzled by the switch to the log scale and wonder about the reasoning for that decision.

Response: We switched to the log scale to make the data closer to a normal distribution.

Finally, I suggest that the authors include (in Supplemental Materials) a table with the values for H19 and Igf1r for each sample.

Response: In response to this suggestion, we have modified Supplementary Table 1 to include the values for both H19 and Igf1r.

I also think they might want to look at the Control and Endo samples separately. The Endo samples are low for both and may be confounding a fair search for correlation. It actually looks to me that in control data points, high H19 does correlate with relatively high Igf1r but that high Igf1r does not predict much about the H19 levels suggesting multiple forms of regulation in controls. Overall, the R value is not very high, probably because multiple things are going on. I think it would make a stronger paper to address this more clearly.

Response: In response to these very insightful comments, we have added new discussion to stress the point that other regulatory mechanisms are likely involved in the H19/Igf1r pathway. Please see page 13, first paragraph, last sentence highlighted in red.

2. It looks like the data in Figure S1 are normalized to H19 levels in cell line 168. Is that correct? I think it will be much more informative to normalize data the same way as in Figure 1 so that the reader can compare the levels of H19 in these cells relative to that seen in the patient and control samples as described in Figure 1. Right now, I have no idea whether the levels of gene expression in the cell lines are similar to those in vivo or are 100-fold higher or lower, for example.

Response: We understand why these questions were raised. We apologize for not being clear in describing the relationship between our in vivo and in vitro data in our previous submission. In response to these questions, we have added a new paragraph in the Discussion. Please see last paragraph on page 13, highlighted in red.

3. Are cell lines 168, 98, 166, and 80 derived from patients from the experiments in Figure 1? If so, can the values for the H19 levels in tissue be included in Figure S1 so that I can assess for myself how well the in vivo and in vitro results correlate?

Response: They were not derived from the patients shown in Figure 1. We understand the points of this reviewer. Please refer to the last paragraph on page 13, highlighted in red for detailed explanation.

4. The authors described several caveats associated with the use of the in vitro system but in the end they make a strong and convincing case for using cell culture models to addressing molecular mechanisms.

Response: We greatly appreciate this comment, which should help to address all the concerns raised in points #2 and #3 above.

5. The experiments described in Figure 2 (A-D) are missing an important control. To address the critical question of whether H19 is working primarily through let7, the essential control data are actually the levels of expression of H19 and Igf1r in cells treated with siCon + iLet7. That is, the question posed by the authors is whether there is any additional effect of H19 once you take let7 regulation of the picture. This siCon + iLet& control is critical for getting some sense of the relative importance of the H19/let7 interactions.

Response: We apologize for not being explicit on this matter in our previous submission, which, we believe, had led to the suggestion of including the siCon + iLet7 group. H19 functions to reduce the bioavailability of let-7 by acting as a molecular sponge (Kallen et al, 2013;Yan et al, 2014;Gao et al, 2014). H19 contains multiple let-7-binding sites that act to sequester let-7 and prevent it from binding to its target mRNA. In other words, it is the bioavailability of let-7 (hence the relative expression levels between H19 and let-7) and NOT its absolute expression level, which determines let-7 target gene expression. Thus, it is important that experiments were done in the context of H19 to clearly demonstrate the relationship between H19 and let-7. Similar experimental designs (i.e., without including the siCon + iLet7) have been used to demonstrate the H19/let-7 axis in both muscle and tumour cells (Yan et al, 2014;Gao et al, 2014). Therefore, we did not include siCon + iLet7 experiments in this paper because they would not add any useful information to the paper, and could even confuse readers. In response to this comment, we have added new sentences highlighted in red on page 9 to be more explicit.

6. Which cell line was used for the experiments described in Figure 2? Do the authors imagine they might see different results (especially in panels E-H) if they used cell lines with relatively high vs relatively low levels of H19.

Response: Thanks much for raising this very important point. In the revised manuscript (also in response to reviewer #2) we show results derived from four different patient cells, with high and low endogenous H19 expression in each group. We used the H19 high cells in our knockdown experiments and H19 low cells in our overexpression experiments. The rationale for using the H19 low for overexpression studies was to avoid potential overexpression artefacts. Please see page 10, first paragraph highlighted in red for detailed explanation.

7. Would there be value in including direct measures of let7 for the studies described in Figure 2.

Response: No, there will be no added values for measuring the let-7 levels. Please refer to our response to point #5 above.

The paper is very well written so my editorial comments are minor:

1. On page 10, "These results suggested that an elevated level of H19 led to reduced bioavailability of let-7..." The experiments are consistent with this but they do not actually address this question. Maybe you could say: "These results are consistent with..."

Response: Thank you. Fixed as suggested. Please see page 11, second paragraph, line 257.

<u>Referee #2</u>

The manuscript of Ghazal and co-authors detail experiments performed using endometrium gathered from women with and without endometriosis. The use of established molecular techniques and primary cell cultures from tissues of affected individuals increases the translational impact of this research. I do have a few concerns and comments which I feel should be addressed before publication.

Supplemental Table 1: Please include average age/BMI +/-SEM for all treatment groups within the body of the Methods section.

Response: Fixed as suggested. Please see revised Supplementary Table 1.

Overall, there is quite a lot of discussion presented in the Methods section (i.e. lines 207-218), which would be better presented in the Discussion to improve clarity of the Results section.

Response: Thanks much for the suggestion. We have moved the referred section to the Discussion and also expended it a bit to be more explicit on the point we wished to make. Please see page 13, last paragraph, highlighted in red in the Discussion.

Line 207: Expression changes in H19 from tissue collection to culture should be confirmed by either PCR or in situ hybridization to confirm results presented in Figure 1; otherwise this could be due to increased numbers of individuals within analyses rendering previous data insignificant. There appears to be a wide range of H19 expression within "normal" control women presented in Figure 1A, this may extend into the affected population when increased numbers are examined.

Response: We understand why these questions were raised, and we apologize for not being clear in our initial submission. As described on page 13, last paragraph in the revised Discussion, we did not observe a statistically significant difference in *H19* expression in cultured endometrial stromal cells from between women with and without endometriosis, and we explained why. We believe that the discrepancy between our in vivo and in vitro results were due to cell culture manipulation. Nonetheless, this in vitro model has enabled us to perform proof-of-principle studies, providing mechanistic insights into how the H19/Let-7/IGFR1 regulatory axis might be working in the stromal cells to impact cell proliferation. Please refer to page 13, last paragraph highlighted in red for a more detailed explanation.

Line 229: You should not "cherry pick" which results to present; otherwise instead of presenting data from three replicates you are presenting one replicate. Normalize data to changes in baseline expression to decrease variation between patients, and consult a statistician to help with the analyses. This is a common practice for data generated from studies in higher species where individual variation is high, unlike those in rodent models where similar genetic background controls variability.

Response: We appreciate this reviewer's concern that the H19/let-7/IGFR1-mediated regulation shown in Figs. 2 and 3 could be patient-specific rather than a general phenomenon of endometrial stromal cells, which was exactly our initial concern as well. To rule out this possibility, we performed H19 knockdown and overexpression experiments in endometrial stromal cells derived from multiple patients and observed results that were consistent with our conclusion. To eliminate possible further confusion to readers, in the revised manuscript, we present data from four different patient cells. Specifically, we performed H19 knockdown experiments in two patient cells that expressed high levels of endogenous H19, and overexpression experiments in other two patient cells with relatively low endogenous levels of H19. The rationale for selecting low H19 cells for overexpression was to avoid possible overexpression-induced artefact. We present results from replicates with statistics in the new Figures 2 and 3, and results from individual cells of the four patients in new Supplementary Figures S2 and S3. Please see page 10, first paragraph red highlighted for detailed explanation.

Figure 2C vs 2G: By Western blot, your controls in 2C express IGF1R, but the empty vector control in 2G does not express IGF1R. You should present data from all replicates to convince me that your protein expression results are a result of the H19 knockdown/ over expression.

Response: Thanks much for pointing this out. We apologize for not being explicit in our previous submission. We used patient cells that expressed low endogenous H19 for the overexpression studies, and this was why the IGF1R signal in Fig. 2G was much weaker than that in Fig. 2C. This is consistent with regulation of IGF1R by H19. Please see page 11 first paragraph highlighted in red.

References

Gao Y, Wu F, Zhou J, Yan L, Jurczak MJ, Lee HY, Yang L, Mueller M, Zhou XB, Dandolo L, Szendroedi J, Roden M, Flannery C, Taylor H, Carmichael GG, Shulman GI, Huang Y (2014) The H19/let-7 double-negative feedback loop contributes to glucose metabolism in muscle cells. *Nucleic Acids Res* 42(22):13799-811.

Kallen AN, Zhou XB, Xu J, Qiao C, Ma J, Yan L, Lu L, Liu C, Yi JS, Zhang H, Min W, Bennett AM, Gregory RI, Ding Y, Huang Y (2013) The imprinted H19 lncRNA antagonizes let-7 microRNAs. *Mol Cell* 52(1):101-12.

Yan L, Zhou J, Gao Y, Ghazal S, Lu L, Bellone S, Yang Y, Liu N, Zhao X, Santin AD, Taylor H, Huang Y (2014) Regulation of tumor cell migration and invasion by the H19/let-7 axis is antagonized by metformin-induced DNA methylation. *Oncogene* doi: 10.1038/onc.2014.236.

2nd Editorial Decision 26 May 2015

Thank you for the submission of your revised manuscript to EMBO Molecular Medicine. We have now received the enclosed reports from the referees that were asked to re-assess it. As you will see the reviewers are now globally supportive and I am pleased to inform you that we will be able to accept your manuscript pending the following final amendments:

1) Please address the comments of referee 1 by tuning down the conclusions in response to point 1. Regarding point 2, we will not ask you to perform the suggested experiments for publication. However, should you have additional data at hand, I would strongly suggest that you include it. In all cases, please discuss appropriately the contribution or not of let-7.

Please submit your revised manuscript within two weeks. I look forward to seeing a revised form of your manuscript as soon as possible.

***** Reviewer's comments *****

Referee #1 (Remarks):

The authors have made important changes that have very significantly improved the quality of the manuscript.

As I stated in my original review, this is an interesting and important study.

Based on their changes, I have two additional comments.

1. The changes to Figure 1C helped clarify the data. To me, these data overall are consistent with the fact that both H19 and Igf1R are decreased in endo patients. (And these data are strong and important). But I think the authors are over interpreting to say there is any apparent correlation between H19 and Igf1R levels. If you consider the two groups separately, there is probably no correlation and therefore no additional information in 1C.

2. In regards to the usefulness for additional controls in Figure 3. There are two possible models for H19 action: it can work through let7 or it can be independent of let7. The authors support model 1 based on previous studies in other model systems. The additional experiment (using iLet7 in both H19 knockdown and H19 overexpression) would allow them to give experimental support for their preferred model in this system. Because I think this is a strong study and addresses a biomedically important topic, I do think that including these additional experiments would be very useful.

Unless I misunderstand Figure 3A, the authors are able to knockdown let7 very effectively (essentially to 0 or at least to an amount that is not statistically different than 0). Therefore it would informative to know whether H19 knockdown or H19 overexertion can have any additional effect in context of no let7. If H19 continues to alter Igf1R levels when let7 is gone, then the authors would need to rethink their molecular model. If H19 levels have no effect in a let7 null background, then this is great experimental support for the authors' model. I think either result is equally important and could potentially impact design for therapeutic approaches.

If it is not feasible to perform these experiments, then the paper should make it even clearer what has and has not been actually tested.

Referee #2 (Remarks):

Thank you for addressing all reviewer concerns. The addition of the supplemental data for all specimens analyzed clarifies the data.

2nd Revision - authors' response 27 May 2015

Point-by-point response to referees' comments (referees' comments in italic)

Referee #1

The authors have made important changes that have very significantly improved the quality of the manuscript.

As I stated in my original review, this is an interesting and important study.

Based on their changes, I have two additional comments.

1. The changes to Figure 1C helped clarify the data. To me, these data overall are consistent with the fact that both H19 and Igf1R are decreased in endo patients. (And these data are strong and important). But I think the authors are over interpreting to say there is any apparent correlation between H19 and Igf1R levels. If you consider the two groups separately, there is probably no correlation and therefore no additional information in 1C.

Response: In response to these comments, we have tuned down the conclusions by using the words "suggested" and "implicating". Please see the red highlighted sentence on page 5 in the revised manuscript.

2. In regards to the usefulness for additional controls in Figure 3. There are two possible models for H19 action: it can work through let7 or it can be independent of let7. The authors support model 1 based on previous studies in other model systems. The additional experiment (using iLet7 in both H19 knockdown and H19 overexpression) would allow them to give experimental support for their preferred model in this system. Because I think this is a strong study and addresses a biomedically important topic, I do think that including these additional experiments would be very useful.

Unless I misunderstand Figure 3A, the authors are able to knockdown let7 very effectively (essentially to 0 or at least to an amount that is not statistically different than 0). Therefore it would informative to know whether H19 knockdown or H19 overexertion can have any additional effect in context of no let7. If H19 continues to alter Igf1R levels when let7 is gone, then the authors would need to rethink their molecular model. If H19 levels have no effect in a let7 null background, then this is great experimental support for the authors' model. I think either result is equally important and could potentially impact design for therapeutic approaches.

If it is not feasible to perform these experiments, then the paper should make it even clearer what has and has not been actually tested.

Response: Thanks much for these very insightful and constructive discussions. In Figure 3, we did

not do let-7 knockdown. But in Figure 2, we did let-7 inhibition by using iLet-7, which is a chemically modified oligonucleotide that specifically binds to let-7 and blocks its function without significantly degrading it. It seems that this reviewer might actually mean Figure 2 and not Figure 3. We apologize for not describing iLet7 clearly, which may have led to the confusion to this reviewer. To avoid further possible confusions to readers, we have added a new sentence highlighted in red on page 6 to clearly define iLet7. As the expression of Igf1r could be fully restored through blocking let-7 by iLet7 in the context of H19 knockdown at both mRNA and protein levels (Figure 2A-D), the contribution of let-7 to the H19-mediated pathway is strongly supported. Please see red-highlighted sentence on page 7 in the revised manuscript.

Referee #2

Thank you for addressing all reviewer concerns. The addition of the supplemental data for all specimens analysed clarifies the data.

Response: Thank you very much!
